# Curcumin Mitigates Immune-Induced Epithelial Barrier Dysfunction by *Campylobacter jejuni*

**DOI:** 10.3390/ijms20194830

**Published:** 2019-09-28

**Authors:** Fábia Daniela Lobo de Sá, Eduard Butkevych, Praveen Kumar Nattramilarasu, Anja Fromm, Soraya Mousavi, Verena Moos, Julia C. Golz, Kerstin Stingl, Sophie Kittler, Diana Seinige, Corinna Kehrenberg, Markus M. Heimesaat, Stefan Bereswill, Jörg-Dieter Schulzke, Roland Bücker

**Affiliations:** 1 Institute of Clinical Physiology/Nutritional Medicine, Medical Department, Division of Gastroenterology, Infectiology, Rheumatology, Charité–Universitätsmedizin Berlin, 12203 Berlin, Germany; fabia.lobo-da-fonseca@charite.de (F.D.L.d.S.); eduard.butkevych@charite.de (E.B.); praveen-kumar.nattramilarasu@charite.de (P.K.N.); anja.fromm@charite.de (A.F.); roland-felix.buecker@charite.de (R.B.); 2 Institute of Microbiology, Infectious Diseases and Immunology, Charité–Universitätsmedizin Berlin, Campus Benjamin Franklin, 14195 Berlin, Germany; soraya.mousavi@charite.de (S.M.); markus.heimesaat@charite.de (M.M.H.); stefan.bereswill@charite.de (S.B.); 3 Medical Department, Division of Gastroenterology, Infectiology and Rheumatology, Charité–Universitätsmedizin Berlin, 12203 Berlin, Germany; verena.moos@charite.de; 4 German Federal Institute for Risk Assessment (BfR), Department of Biological Safety, National Reference Laboratory for Campylobacter, 12277 Berlin, Germany; julia.golz@bfr.bund.de (J.C.G.); kerstin.stingl@bfr.bund.de (K.S.); 5 University of Veterinary Medicine Hannover, Research Center for Emerging Infections and Zoonoses, 30559 Hannover, Germany; sophie.kittler@tiho-hannover.de (S.K.); diana.seinige@tiho-hannover.de (D.S.); 6 Institute for Veterinary Food Science, Justus-Liebig-University, 35392 Giessen, Germany; corinna.kehrenberg@vetmed.uni-giessen.de

**Keywords:** *Campylobacter jejuni*, curcumin, tight junction, claudin, apoptosis, co-culture, mouse colon, cytokines, TNF, NFκB

## Abstract

*Campylobacter jejuni* (*C. jejuni*) is the most common cause of foodborne gastroenteritis worldwide. The bacteria induce diarrhea and inflammation by invading the intestinal epithelium. Curcumin is a natural polyphenol from turmeric rhizome of *Curcuma longa*, a medical plant, and is commonly used in curry powder. The aim of this study was the investigation of the protective effects of curcumin against immune-induced epithelial barrier dysfunction in *C. jejuni* infection. The indirect *C. jejuni*-induced barrier defects and its protection by curcumin were analyzed in co-cultures with HT-29/B6-GR/MR epithelial cells together with differentiated THP-1 immune cells. Electrophysiological measurements revealed a reduction in transepithelial electrical resistance (TER) in infected co-cultures. An increase in fluorescein (332 Da) permeability in co-cultures as well as in the germ-free IL-10^−/−^ mouse model after *C. jejuni* infection was shown. Curcumin treatment attenuated the *C. jejuni*-induced increase in fluorescein permeability in both models. Moreover, apoptosis induction, tight junction redistribution, and an increased inflammatory response—represented by TNF-α, IL-1β, and IL-6 secretion—was observed in co-cultures after infection and reversed by curcumin. In conclusion, curcumin protects against indirect *C. jejuni*-triggered immune-induced barrier defects and might be a therapeutic and protective agent in patients.

## 1. Introduction

*Campylobacter jejuni* is the most prevalent pathogenic bacterium of zoonotic gastroenteritis [1]. Typical symptoms provoked by *C. jejuni* are watery to bloody diarrhea, abdominal pain, fever, and nausea [2]. This human pathogen is present in the intestinal microbiota of farm animals, especially poultry, which is the main source of infection for humans by ingestion of contaminated or undercooked food [1]. The bacteria adhere to the mucus and the surface of intestinal epithelial cells, invade the intestinal epithelium, while they pass the cells via the transcellular or paracellular route [3,4]. Consequently, direct epithelial barrier defects such as dysregulation of tight junction (TJ) proteins, induction of epithelial lesions or also indirect effects by inflammatory responses of epithelial or immune cells occur [5,6].

Epithelial lesions can go along with single-cell lesions by increased apoptosis, mid-sized leaks like focal leaks, as well as erosions or even ulcerations. These pathological findings could explain the type of diarrhea for the *Campylobacter* infection as leak flux pathomechanism, which is characterized by a loss of water and solutes from the organism into the intestinal lumen through a leaky epithelium [6,7]. For a physiological intestinal integrity, an intact epithelium is essential. The main components of intestinal epithelial barrier are the TJs. They are the apical components of the intercellular seal and divide the epithelial cell membrane into an apical and basolateral compartment (fence function) [8]. Tight junctions form a barrier for water, ions, and solutes (barrier function) [9] and consist of various molecular transmembrane proteins like claudins and occludin, junction adhesion molecules (JAM), tricellulin, and cytoplasmatic scaffolding proteins as zonula occludens protein-1 (ZO-1) [8,9].

Recently, the number of *C. jejuni* infections surpasses *Salmonella* infections as the leading cause of zoonotic bacterial gastroenteritis in the US and Europe. Moreover, high levels of fluoroquinolone resistance have been reported [10] and levels of multi drug resistances (MDR) are increasing [11]. In consequence, new therapeutic targets as well as innovative strategies to reduce the amount of *C. jejuni* in farm animals and environment are urgently required. A potent natural substance thought to interfere with *Campylobacter* infection is curcumin.

Curcumin is a polyphenolic compound found in the turmeric root of the *Curcuma longa* plant (commonly known as turmeric), a member of the ginger family [12,13,14]. It is used as coloring agent, cosmetics, and it is the principal ingredient in oriental food spice like curry powder [12,13]. For centuries, curcumin has been used in Asian traditional medicine as a common nontoxic and active agent against different gastrointestinal and digestive disorders [12,14,15]. In addition, curcumin is known to possess anti-inflammatory, antioxidative, antibacterial, anticarcinogenic [16], antiviral [15,17], antiapoptotic, and antiproliferative properties [12]. The antibacterial effect was shown against *Salmonella* spp. [18], *Helicobacter pylori* [16,19,20], methicillin-resistant *Staphylococcus aureus*, *Bacillus subtilis*, and *Escherichia coli* [21]. Various studies have shown that curcumin modulates inflammatory cytokine pathways like IL-1β, TNF-α, IL-6, IL-8, IL-10 [12,13,22,23], and inhibit signaling pathways like NFκB [12,17,19].

The aim of the presented study was to evaluate the barrier-protective and anti-inflammatory properties of curcumin on intestinal epithelial barrier function, which is compromised by the *C. jejuni*-mediated immune response. Therefore, a novel in vitro co-culture model consisting of intestinal epithelial HT-29/B6-GR/MR and immune THP-1 cells was established. This co-culture model enables the screening of potential barrier-protective and anti-inflammatory compounds in *C. jejuni* infection, as a result of which curcumin was identified as being effective.

## 2. Results

### 2.1. Establishment of a Co-Culture with Colon Epithelial Cells HT-29/B6-GR/MR and Immune THP-1 Cells

**Figure 1:**
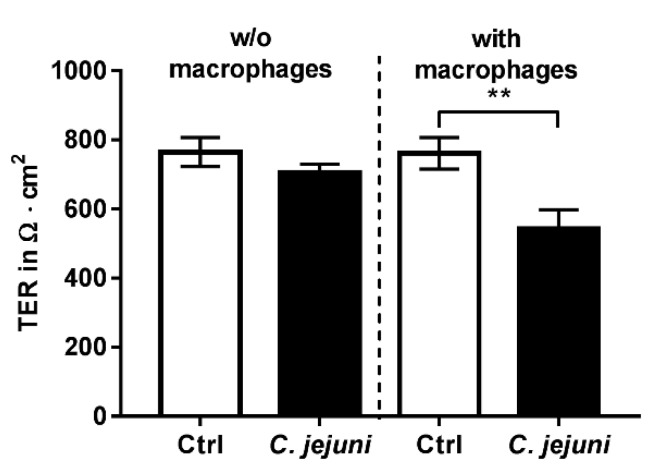
*C. jejuni* infection in human intestinal epithelial cells (HT-29/B6-GR/MR) or in co-culture together with human immune cells (differentiated THP-1 cells). Transepithelial electrical resistance (TER) in co-culture after *C. jejuni* infection from the basal side decreased 48 h post infection (*n* = 7–9, ** *p* < 0.01, unpaired Student’s *t*-test).

To analyze the impact of the immune response during a *Campylobacter* infection on epithelial barrier function, human colon epithelial cells and immune cells were co-cultured. Colonic epithelial cells (HT-29/B6-GR/MR) were seeded on cell culture filter supports (0.4 µm pore size) and represented the epithelial compartment. Under the membrane of the epithelial cell filter, THP-1 immune cells were seeded on the bottom of the cell culture well and represented the subepithelial compartment. Basal bacterial infection with *C. jejuni* decreased transepithelial electrical resistance (TER) when colonic epithelial and immune cells (differentiated THP-1 cells) were co-cultured (Figure 1). From the apical supernatant, no bacteria could be cultured after the experiment, indicating that the bacteria could not pass the filter membrane with 0.4 µm pore size. As a result of this, and in contrast to the results in the co-culture model, *C. jejuni* infection at the basal membrane had no effect on TER in HT-29/B6-GR/MR mono-culture (Figure 1).

### 2.2. Curcumin Improves Disturbed Intestinal Barrier Function in the Co-Culture System

**Figure 2:**
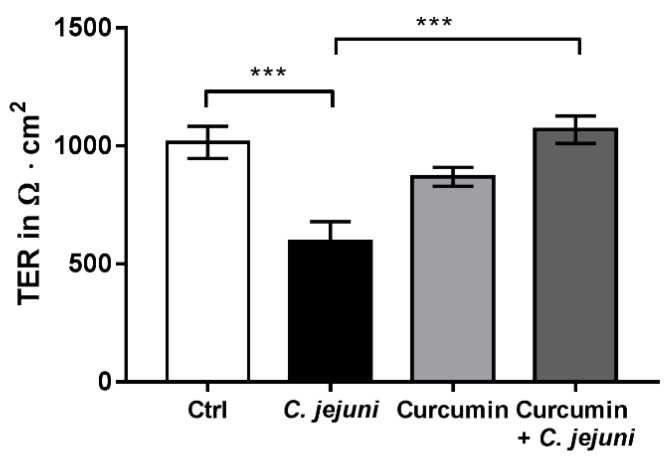
Curcumin protects against *C. jejuni*-induced barrier disruption in co-culture. Cells were treated with 50 µM curcumin and infected with *C. jejuni* for 48 h (*n* = 13, *** *p* < 0.001, one-way ANOVA with Bonferroni correction for multiple comparisons).

Using mRNA sequencing and subsequent ingenuity pathway analysis (IPA) of human mucosa biopsies from *C. jejuni*-infected patients, inhibition of curcumin-dependent pathways indicated that the presence of curcumin might prevent the *C. jejuni*-induced changes in gene expression. Based on this bioinformatic prediction of curcumin’s effect on host gene expression, the addition of curcumin should activate downstream pathways and might reduce the effects of *Campylobacter* infections (Supplementary Table S1). In order to test the barrier protective and anti-inflammatory properties of curcumin in *Campylobacter*-induced barrier dysfunction, a curcumin solution was added to the infected co-culture model. As shown in Figure 2, *C. jejuni* reduced TER significantly 48 h after infection, whereas this change was inhibited by the presence of curcumin.

**Figure 3:**
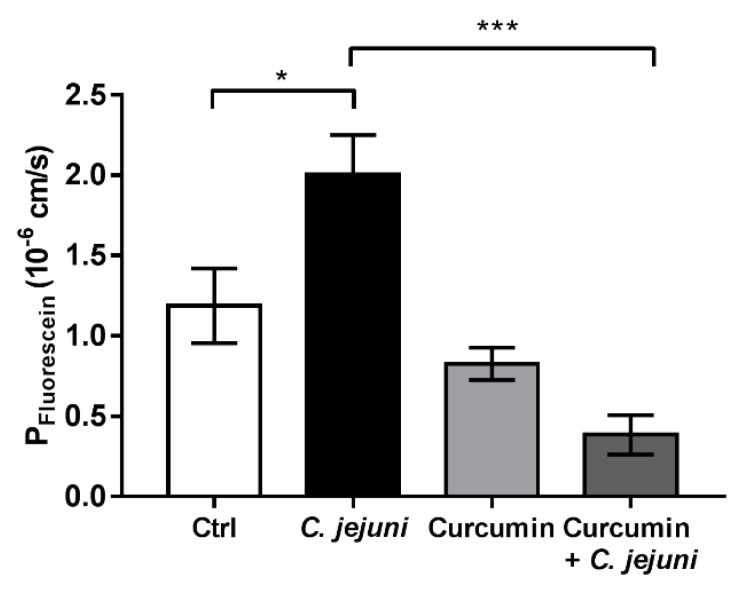
Curcumin improves the *C. jejuni*-induced increase in permeability to fluorescein (332 Da) in co-cultures with colon epithelial HT-29/B6-GR/MR and immune THP-1 cells. The co-cultures were infected with *C. jejuni* after incubation with 50 µM curcumin. Fluorescein permeabilities were measured 48 h post infection (*n* = 7–8, * *p* < 0.05, *** *p* < 0.001, one-way ANOVA with Bonferroni correction for multiple comparisons).

Permeability studies were performed with the paracellular flux marker fluorescein (332 Da). Compared to controls, *C. jejuni* infection caused an increase in permeability to fluorescein. Application of curcumin not only ameliorated the permeability increase for fluorescein, but even resulted in an improved barrier function for paracellular passage of macromolecules when exceeded the control level (Figure 3).

### 2.3. Effect of Curcumin In Vivo on Intestinal Barrier Regulation in IL-10^−/−^ Mice

**Figure 4:**
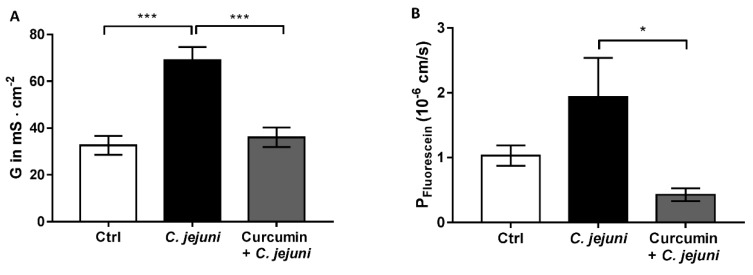
Curcumin ameliorates the *C. jejuni*-induced barrier dysfunction in colon of IL-10^−/−^ mice. Colon specimens of IL-10^−/−^ mice were mounted in Ussing chambers designed for impedance spectroscopy. (**A**) Epithelial conductance (G) (*n* = 5–18, *** *p* < 0,001, Mann–Whitney *U*-test) and (**B**) fluorescein (332 Da) permeability (*n* = 5–14, * *p* < 0.05, Mann–Whitney *U*-test) of IL-10^−/−^ mouse colon were measured in Ussing chambers. IL-10^−/−^ mice were either infected with *C. jejuni* (per oral gavage with 10 ^8^ CFU in 0.3 mL) or infected and treated for 6 days with Curcumin (0.5 mg/mL).

Secondary abiotic IL-10^−/−^ mice were infected with *C. jejuni* and treated with 0.5 mg/mL of curcumin, starting 4 days prior infection until the end of the observation period (i.e., day 6 post infection). Epithelial barrier function of distal colon specimens from IL-10^−/−^ mice was analyzed in Ussing chambers. Conductance (G, Figure 4A) and permeability to fluorescein (Figure 4B) was significant higher in *C. jejuni*-infected mice than in untreated infected mice. Mice receiving curcumin showed a significantly lower conductance and permeability to fluorescein than untreated infected mice. The electrophysiological results obtained in vivo (Figure 4) resemble the results from the in vitro co-culture system (Figure 3).

### 2.4. Effect of Curcumin on Bacterial Integrity

To determine whether curcumin had a direct effect on the viability of *C. jejuni* in the concentration used in our experiments, the minimal inhibitory concentration (MIC) for curcumin was determined. The MIC of curcumin in *C. jejuni* 81–176 amounted to 87 µM under physiological conditions at a pH of 7.4, and is thus above our experimental curcumin concentrations in the co-culture. Moreover, if curcumin is used as treatment, it is necessary to know whether the substance exerts any stress to the bacterium, leading to enhanced adaption by, e.g., stimulation of natural transformation capacity. Therefore, competence development and DNA uptake of *C. jejuni* was investigated under the treatment of curcumin using a single cell assay. After curcumin treatment, no change in the ratio of competent bacteria was registered (control 26% ± 2% versus treated 25.5% ± 0.5% with 50 µM curcumin, *n* = 3, n.s., unpaired Student’s *t*-test). In addition, also the DNA uptake processes in competent *C. jejuni* was unaffected after curcumin treatment (control 100% versus treated 86.9% ± 6.7%, *n* = 3, n.s., unpaired Student´s *t*-test).

### 2.5. Cytokine Secretion in C. jejuni-Infected Co-Cultures

**Figure 5:**
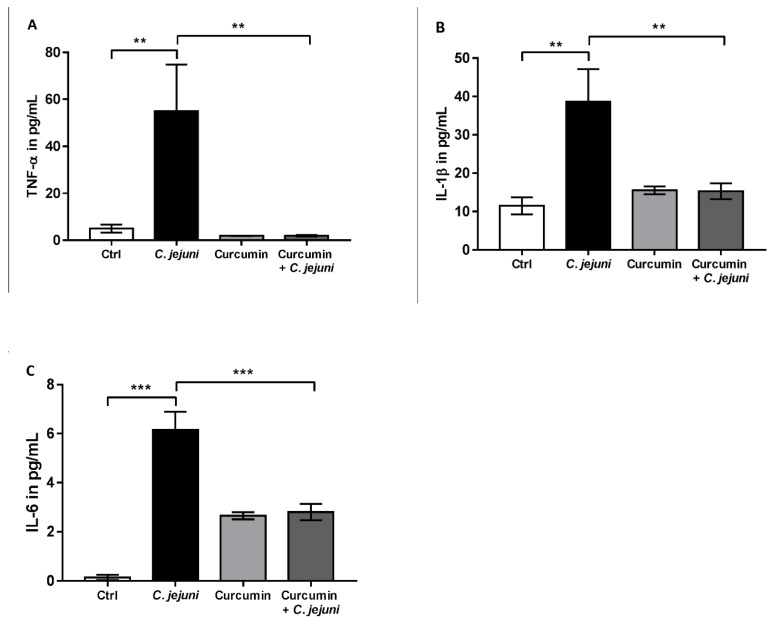
Cytokine release in co-cultures after *C. jejuni* infection. (**A**) Tumor necrosis factor-α (TNF-α), (**B**) Interleukin-1β (IL-1β), and (**C**) Interleukin-6 (IL-6) secretion after treatment with curcumin in *C. jejuni*-infected co-cultures (*n* = 5–6, * *p* < 0.05, ** *p* < 0.01, *** *p* < 0.001, one-way ANOVA with Bonferroni correction for multiple comparisons).

To identify which cytokines were released by the immune cells and affected epithelial cells function, cytometric bead arrays (CBA) from supernatants of treated co-cultures were performed. TNF-α, IL-1β, and IL-6 were increased in *C. jejuni*-infected co-cultures, while this changes were prevented by curcumin treatment (Figure 5).

### 2.6. NFκB Signaling Pathway is Involved in *C. jejuni*-Induced Barrier Dysfunction

**Figure 6:**
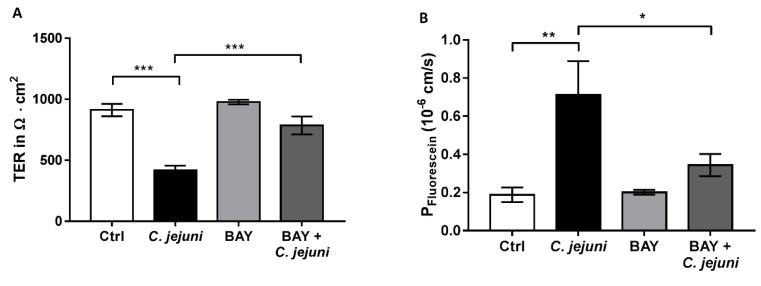
The NFκB pathway is barrier-relevant. Inhibitory effect of BAY 11-7082 in *C. jejuni*-infected co-cultures. (**A**) Transepithelial electrical resistance (TER) and (**B**) permeability to fluorescein (332 Da) after incubation with 10 µM BAY 11-7082 and infection with *C. jejuni* for 48 h (*n* = 5, * *p* < 0.05, ** *p* < 0.01, *** *p* < 0.001, one-way ANOVA with Bonferroni correction for multiple comparisons).

Furthermore, we evaluated the possible relevance of the NFκB pathway for barrier function in our cell model using the NFκB inhibitor BAY 11-7082 (BAY). BAY inhibited the *C. jejuni*-induced reduction in TER, similar to the effect of curcumin (Figure 6A). In addition, the increase in permeability to fluorescein could be reversed after BAY inhibition and almost reached control values (Figure 6B).

### 2.7. Epithelial Apoptosis is Blocked by Curcumin in the Co-Culture Model

**Figure 7:**
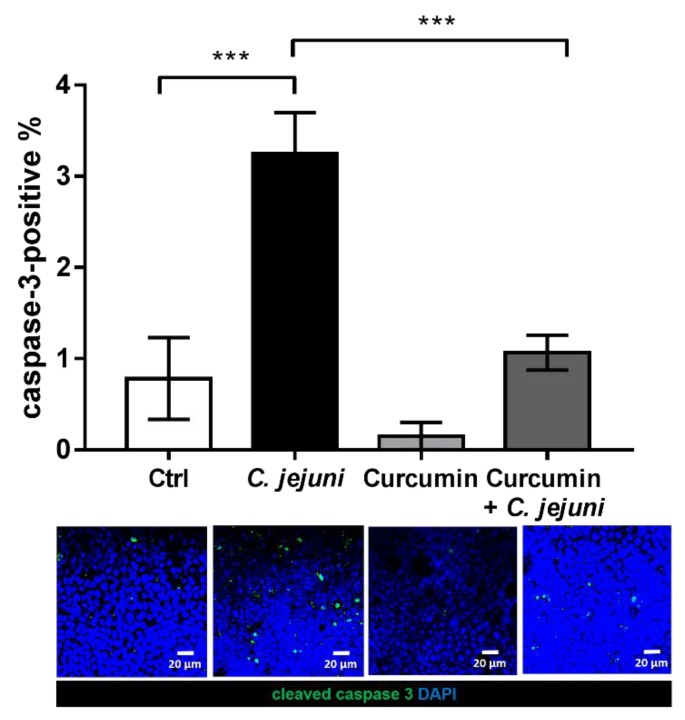
Apoptosis induction in *C. jejuni*-infected co-cultures. Cleaved caspase-3 staining indicates the number of apoptotic cells and is visualized by confocal microscopy. Monolayers were stained with antibodies against cleaved caspase-3 (green) and nuclei were colored with 4´-d-diamidino-2-phenylidole dihydrochloride (DAPI, blue). Signals of cleaved caspase-3-positive cells in CLSM were counted in the top view pictures and related to the number of DAPI stained nuclei (~300 nuclei/frame) to calculate the percentage of apoptotic cells in the monolayers (*n* = 6, *** *p* < 0.001, one-way ANOVA with Bonferroni correction for multiple comparisons). Representative pictures are shown for HT-29/B6-GR/MR cells, obtained from co-culture after treatment with 50 µM curcumin and infection with *C. jejuni*.

Given that the induction of epithelial apoptosis constitutes a barrier-relevant pathomechanism, and *Campylobacter* infections have been shown to induce epithelial apoptosis, the impact of curcumin on apoptosis was analyzed using cleaved caspase-3 staining and visualized by confocal laser-scanning microscopy (CLSM) (Figure 7).

After infection with *C. jejuni*, the apoptotic ratio was increased 3-fold in comparison to control. Incubation with curcumin during the infection with *C. jejuni* reduced the apoptotic ratio to that of controls (Figure 7) (control 0.7% ± 0.4% versus infected 3.3% ± 0.4% versus treated 1.1% ± 0.2%, *n* = 6, ** *p* < 0.01, *** *p* < 0.001, one-way ANOVA with Bonferroni correction for multiple comparisons).

### 2.8. Influence of *C. jejuni* and Curcumin on Tight Junction Protein Expression in the Co-Culture Model

**Figure 8:**
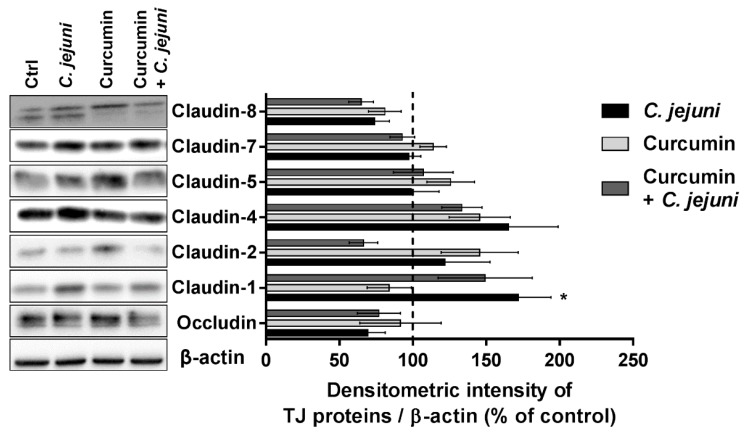
Tight junction (TJ) protein expression in western blot. Representative western blots and densitometry of TJ protein expression from HT-29/B6-GR/MR cells obtained from co-culture after incubation with 50 µM curcumin and infection with *C. jejuni*. TJ proteins were normalized to the level of β-actin (*n* = 6–9, * *p* < 0.05, two-way ANOVA with Bonferroni correction for multiple comparisons. Control value is marked with a dashed line.

To define the *C. jejuni*-induced tight junction (TJ) expression changes, protein lysates were generated from colon epithelial cells of co-cultures after treatment with curcumin and infection with *C. jejuni* (Figure 8). The expression change was quantified by densitometric analysis with β-actin as loading control, which showed an increase in protein expression of claudin-1 protein expression after *C. jejuni* infection. Claudin-1 expression seemed to decrease again after curcumin treatment in the *C. jejuni* infected group. However, this did not reach statistical significance. No further expression changes of the tested TJ proteins could be observed. A reduction by trend after *C. jejuni* infection in occludin and claudin-8 was detectable, but without reaching statistical significance after multiple test correction (claudin-8 infected versus curcumin-treated *p* = 0.046, uncorrected Student´s *t*-test).

### 2.9. Influence of *C. jejuni* and Curcumin on Subcellular TJ Protein Distribution in Co-Cultures in Confocal Laser-Scanning Microscopy

**Figure 9:**
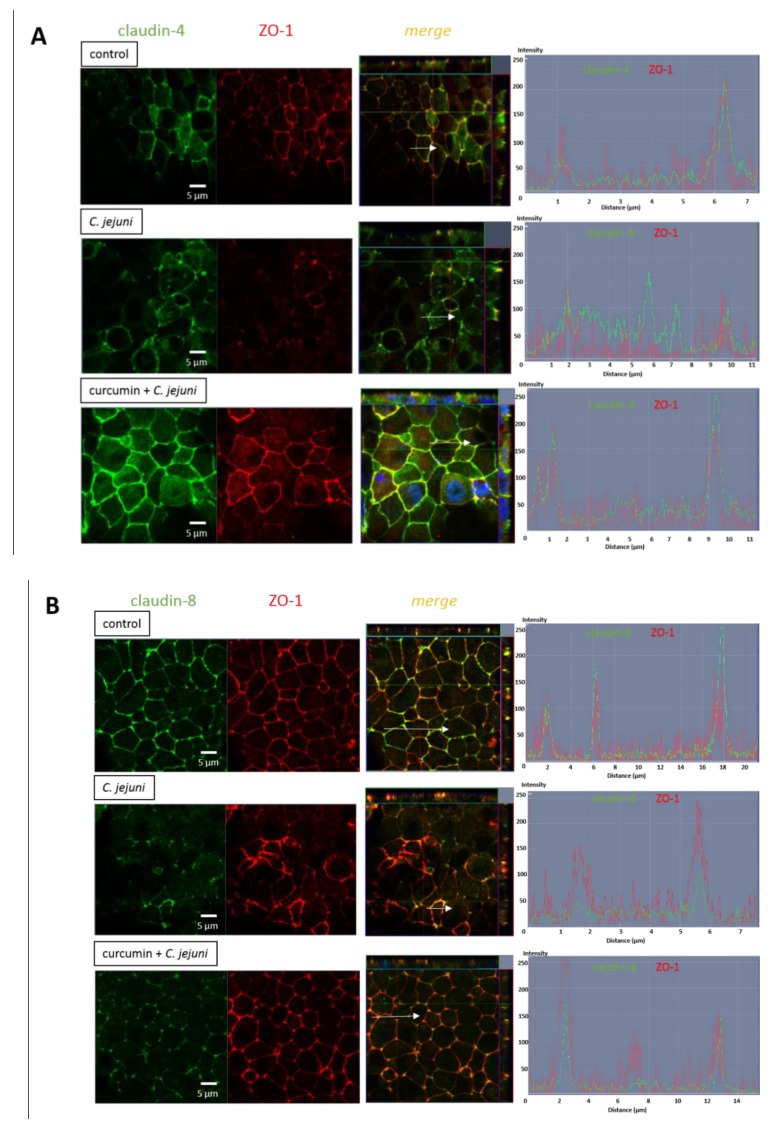
Tight junction distribution in *C. jejuni* infection after treatment with curcumin. Representative confocal laser-scanning microscopy pictures of HT-29/B6-GR/MR after co-culturing together with immune cells. (**A**) Claudin-4 (green) and zonula occludens protein-1 (ZO-1, red), and (**B**) claudin-8 (green) and ZO-1 (red). Nuclei are stained in blue with 4´-6-diamidino-2-phenylindole dihydrochloride (DAPI).

Since the composition and presence of TJ proteins in the TJ strands influence barrier function, the subcellular distribution of the TJs was analyzed with confocal laser-scanning microscopy (CLSM). Z-stacks were generated from different TJ proteins of the immunostained epithelial cell monolayers, and intensity-distance plots were generated. The TJ strands after *C. jejuni* infection indicated a redistribution of claudin-4 (Figure 9A) and claudin-8 (Figure 9B). Claudin-4 and claudin-8 signals were retracted from the TJ strands. Moreover, claudin-4 and claudin-8 were no longer co-localized with zonula occludens protein-1 (ZO-1) after *C. jejuni* infection. Intensity–distance plots with high background intensities indicate re-localization into the intracellular regions. After treatment with curcumin, the appearance of claudin-4 and claudin-8 improved. As a consequence, the infected and curcumin treated group showed co-localization (merging), with high peak intensities in the areas of TJ contact, comparable to the control. Curcumin control groups showed no difference compared to the untreated controls (Supplementary Figure S2).

## 3. Discussion

It is important to contain the *Campylobacter jejuni* infection because of its impact on gastroenteritis worldwide and resulting sequelae, such as irritable bowel syndrome, reactive arthritis, or Guillian–Barré syndrome. Therefore, we aimed to study the underlying pathomechanisms for barrier dysfunction and the influence of the immune system in more detail. For this purpose, we used a new in vitro co-culture model and an in vivo mouse model to study the protective properties of the natural polyphenol curcumin.

Curcumin is a vitamin D receptor (VDR) ligand [24], and we found the downstream signaling pathway of curcumin to be inhibited in the mucosa of acutely infected *C. jejuni* patients by means of the bioinformatic prediction in Qiagen ingenuity pathway analysis (IPA) from RNA-sequencing (Supplementary Table S1). This prediction indicated that curcumin could be a potential therapeutic or preventive target against *C. jejuni* infections. Based on this bioinformatics prediction, we tested the effects of curcumin on *C. jejuni* infection in vitro and in vivo. The inhibitory effect of another top hit upstream regulator in IPA analysis on the mucosa of *C. jejuni*-infected patients was calcitriol (active form of vitamin D). Calcitriol exhibited a high significance value comparable to curcumin (Appendix A, Table S1). Annulment of *C. jejuni*-induced cytotoxicity after calcitriol treatment has been demonstrated in HT-29/B6 cells previously, supporting the view that the bacterium affects intestinal barrier function via a VDR-dependent pathway [6].

As predicted by the pathway analysis in acute human campylobacteriosis, curcumin was effective in our experiments. It could prevent the decrease in TER as well as the increase in fluorescein permeability in our co-culture model, indicating improved barrier function by curcumin in immune-mediated epithelial barrier dysfunction by *C. jejuni*.

Because we expected an essential contribution of the immune system, we established a co-culture model with basal infection. In this model, the *C. jejuni*-triggered immune response reduced the TER after 48 h. The apical bathing medium did not contain bacteria even after 48 h, which suggests that the effect on resistance was caused indirectly by the *C. jejuni*-induced immune response. The decrease in TER may therefore reflected an increase in paracellular permeability secondary to epithelial barrier defects, which would increase permeability to small molecules and toxins. Furthermore, this change in barrier function may increase the access of bacteria to the underlying tissue and potentiate the immune response, causing additional barrier disruption, with loss of cellular integrity [6,25]. Concomitantly, a leak-flux type of diarrhea is caused that represents a main diarrheal mechanism.

Depending on the cell type and the experimental infection conditions, *C. jejuni* was more or less able to modulate TER in previous studies. No effects on TER have been found in MKN-28 cells [26] or Caco-2 monolayers [4]. In contrast, exposure to *C. jejuni* has been reported to reduce TER in T84 and MDCK-I cells [27]. In our HT-29/B6-GR/MR cells, TER was also not different from control even after 48 h basal infection, although a decrease in TER after basolateral infection has been reported to be more pronounced than after apical infection in T84 cells [28]. That this was not seen in our HT-29/B6-GR/MR cells may reflect differences in cell type and/or the 0.4 µm filter pore size of our filters, limiting the bacterial access to the epithelial cells.

Within 6 days following peroral *C. jejuni* infection, secondary abiotic IL-10^−/−^ mice develop acute ulcerative enterocolitis mimicking key features of severe campylobacteriosis in humans, with a specific cytokine response [29]. We were able to show for the first time, using impedance spectroscopy in Ussing chambers, compromised barrier integrity with an increased epithelial conductance and permeability to macromolecules in this mouse model. These data reflect the results of our co-culture model, in which the barrier defects could be similarly prevented by curcumin. Bücker et al. [6] incubated colonic mucosal biopsies from *C. jejuni*-infected patients in culture medium and quantified cytokine release into the supernatant. Secretion of TNF-α, IFN-γ, IL-1β, and IL-13 was enhanced in comparison to controls [6]. It was concluded that this mucosal cytokine storm led to disruption of intestinal TJs and the induction of epithelial apoptosis. In our infected co-cultures, release of cytokines was also mainly responsible for the barrier defects, even though THP-1 cells produce and secrete a lower amount and spectrum of cytokines in comparison to fresh isolated peripheral blood mononuclear cells (PBMC). After *C. jejuni* infection, levels of the pro-inflammatory cytokine TNF-α, IL-1β, and IL-6 increased remarkably, and these changes were largely prevented by curcumin. Curcumin not only reduced local inflammation in the intestine, it also reduced systemic inflammation triggered by LPS [23]. In another study, curcumin nanoparticles suppressed the expression of mRNAs encoding pro-inflammatory mediators, including TNF-α, IL-1β, and IL-6 [14].

*C. jejuni* was found to activate the NFκB signaling pathway [28], which was suppressed by curcumin [12,17,19]. To determine the role of the NFκB pathway in maintaining barrier function, inhibition studies with the NFκB-inhibitor BAY 11-7082 were performed in *C. jejuni*-infected co-cultures. BAY 11-7082 treatment inhibited both the *C. jejuni*-induced decrease in TER and the increase in fluorescein permeability. This points to the involvement of NFκB in the regulation of epithelial barrier function. Similar results were obtained by our group previously with the bioactive ginger ingredient 6-shogaol after TNF-α stimulation in Caco-2 and HT-29/B6 cells [30].

In our co-culture, *C. jejuni* provoked paradoxical upregulation of claudin-1 (Claudin-1 paradox); that is, upregulation of the barrier-forming TJ protein claudin-1 while epithelial resistance was reduced, which we explained by the re-distribution of claudin-1 off the tight junction domain [6]. Claudin-1 induction has also been observed before in human colon biopsies after *C. jejuni* infection [6], *C. fetus* or *C. coli* infection in HT-29/B6 cells [31], and pro-inflammatory cytokine stimulation in HT-29/B6 cells [32,33]. A correlation between increased claudin-1 protein expression and apoptosis induction also occurs in HT-29/B6 cells [34]. In addition, curcumin induced claudin-4 mRNA expression in Caco-2 cells [8]. However, in our co-culture model, curcumin did not induce claudin-4 protein expression. Cell type-dependent differences in TJ expression were also found for quercetin, another plant-derived polyphenol with barrier-improving properties, which increased claudin-4 protein expression in Caco-2, but not in HT-29/B6 cells [35,36].

In order to exclude antibacterial effects of curcumin in our study, the MIC was determined with 87 µM (at pH 7.4). This suggests that the protective effects of 50 µM curcumin were not based on its antibacterial properties. Thus, we conclude that curcumin had direct barrier-protective and anti-inflammatory properties in the host cells.

To further exclude interfering properties of curcumin with bacterial fitness and adaptive response, we investigated if curcumin changes natural transformation capacity. Therefore, we used a single cell DNA uptake assay and challenged the bacteria with 50 µM curcumin. *C. jejuni* is naturally competent for DNA uptake from the environment. Generally, this leads to genetic recombinations between bacterial strains, and as a consequence, to more diversity [37]. Curcumin did not influence the competence development and horizontal DNA uptake of *C. jejuni* in our experimental concentrations.

One of the major challenges to the development of therapeutic approaches is the remarkable diversity between different *Campylobacter* strains in animals, the environment, and food [2]. Consequently, the efficacy of curcumin should also be tested using other *C. jejuni* field strains and, subsequently, in other *Campylobacter* species such as *C. coli* or *C. concisus*. Curcumin is a natural agent, and should be more appropriate than synthetic drugs [12]. Curcumin has been shown to be beneficial in maintaining remission in ulcerative colitis patients when used as a complementary therapeutic substance together with the antiphlogistic compound mesalazine in several randomized clinical studies [38,39,40]. Since the immune response plays an important role in pathogenesis in IBD as well as in *C. jejuni* infection, curcumin may be equally effective in immune-induced barrier dysfunctions by *C. jejuni* as an add-on to other anti-inflammatory drugs. Indeed, multimodal therapies may have an even greater protective effect, and synergism has been reported between curcumin and quercetin [41], genistein [42], epigallocatechin-3-gallate [15,43], and antioxidants [12,44]. Thus, curcumin alone or in combination with other agents may be useful in preventing, treating, and combating *Campylobacter* infection in humans, *C. jejuni*-associated sequelae as well as colonization of farm animals and especially poultry.

Taken together, our results suggest that a significant part of the *C. jejuni*-induced barrier defect is mediated indirectly by the immune cells. Curcumin abolishes functional epithelial disorders as well as pro-inflammatory cytokine secretion. Further barrier-protective or anti-inflammatory compounds, as well as curcumin combinations with other therapies, should be tested in the co-culture model.

## 4. Materials and Methods

### 4.1. Epithelial Cell Culture and Differentiation of THP-1 Cells

**Figure 10:**
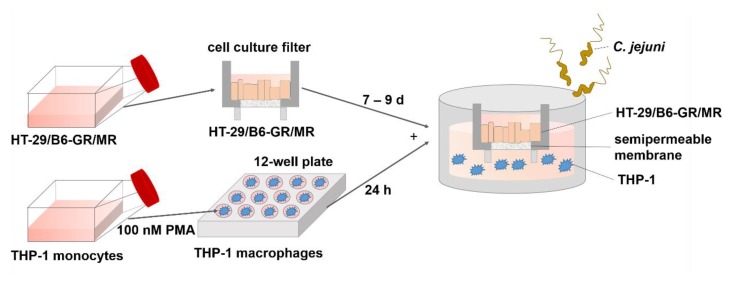
Experimental setting for co-culture of colon epithelial HT-29/B6-GR/MR and THP-1 immune cells. For the continuous culture of epithelial and immune cells, cells were cultured in culture flask. Epithelial cells were then seeded on filter membranes and differentiated over 7–9 days to a polarized monolayer with tight junctions. 24 h before co-culture started, immune THP-1 cells were stimulated with phorbol 12-myristate 13-acetate (PMA) to allow a differentiation to adherent macrophage-like immune cells. After the differentiation of epithelial and immune cells, filters were placed into the 12-well plate with epithelial cells in the apical and immune cells in the basal compartment. Infection with *C. jejuni* was performed in the basal compartment.

The human colon carcinoma cell line HT-29/B6-GR/MR [45], a subclone of the HT-29/B6 line [46] was cultured in 25 cm^2^ culture flasks in RPMI 1640 culture medium (Sigma Aldrich, St. Louis, MO, USA). HT-29/B6-GR/MR cells expressing the human glucocorticoid receptor α and the human mineralocorticoid receptor, were established earlier by stable transfection [45,47]. The culture medium was supplemented with 10% fetal calf serum (FCS; Gibco, Carlsbad, CA, USA), 1% penicillin/streptomycin (Corning, Wiesbaden, Germany), G418-BC (300 µg/mL; Invitrogen, Carlsbad, CA, USA) and hygromycin B (200 µg/mL; Biochrom GmbH, Berlin, Germany), and cells were passaged every week. Cells were seeded on Millicell PCF filters membranes (Merck Millipore, Billerica, MA, USA; 0.4 µm pore size and an effective growth area of 0.6 cm^2^). The cell medium in the culture flask, as well as in the filters, were changed every other day. Experiments were performed between 7 and 9 days after seeding, when polarized cells formed confluent monolayers and the transepithelial electrical resistance was 600–900 Ω∙cm^2^. These cells were chosen because of their stable growth behavior and the high resistance. The human monocyte leukemia cell line THP-1 (ATCC TIB-202) was provided by Verena Moos. THP-1 cells were cultured in RPMI 1640 supplemented with 10% heat-inactivated FCS and 1% penicillin/streptomycin. Cultures were maintained by the addition of fresh medium and were subcultured before reaching a density of 8 × 10^5^ cells/mL once a week. THP-1 cells were re-suspended in antibiotic-free medium supplemented with phorbol 12-myristate 13-acetate (PMA; Sigma Aldrich, St. Louis, MO, USA; solved in DMSO) at a final concentration of 100 nM, seeded with a density of 1.8 × 10^5^ in 12-well plates, and incubated for 24 h for differentiation to macrophages [48]. After incubation, PMA-containing medium was removed and the differentiated and adherent macrophages-like cells were cultured together with epithelial monolayers grown on filters in co-culture (Figure 10). All cells were cultured at 37 °C in a humidified 5% CO_2_ atmosphere.

### 4.2. Growth Conditions of C. jejuni, Treatment and Infection Procedure In Vitro

*Campylobater jejuni* wildtype (wt) 81-176 reference strain was pre-cultured on blood agar plates (Oxoid, Thermo Scientific, Waltham, MA, USA) under microaerobic conditions (5–10% O_2_, 10% CO_2_, 85% N_2_) at 37 °C and re-cultured a second time before infection experiments. Microaerobic conditions were generated in a plastic jar with CampyGen gaspacks from Oxoid (Oxoid, Thermo Scientific, Waltham, MA, USA). Bacteria were then cultured in Mueller–Hinton broth for at least 2.5 h at 37 °C under microaerobic conditions, centrifuged (2 min, 5000 *g*, 10 °C), and re-suspended in the cell culture medium. The optical density at OD_600_ was adjusted to 1 and the cells where infected from basal side with a multiplicity of infection (MOI) of 100. At least 1.5 h before the infection, cells were washed three times with antibiotic-free culture medium supplemented with 10% heat-inactivated FCS. To analyze the barrier-protective and anti-inflammatory properties of curcumin (Sigma Aldrich, St. Louis, MO, USA; final concentration 50 µM), different conditions were tested: control, *C. jejuni* infected, curcumin control (50 µM), and curcumin in combination with *C. jejuni*. Curcumin control showed no adverse effects on the cell viability of HT-29/B6-GR/MR monolayers at the concentration tested. Cells were pre-incubated with curcumin on both sides for 2 h, and infected with *C. jejuni* from the basal side for a direct immune cell infection and cytokine-induced barrier effect. Curcumin was added to the apical and basal side, since functional measurements depend on identical solutions on both the sides of the monolayer to avoid, e.g., potential osmotic effects. Moreover, curcumin readily accessed the basolateral compartment via *C. jejuni*-induced leaks. After infection, cells were incubated at 37 °C under microaerobic conditions favorable to the bacteria for 48 h.

### 4.3. Generation of Secondary Abiotic IL10^−/−^ Mice, Treatment Infection

Permeability to fluorescein (332 Da; Sigma Aldrich, St. Louis, MO, USA) measurements were performed in Ussing chambers with colon of secondary abiotic IL-10^−/−^ mice suffering from acute enterocolitis within 6 days following peroral *C. jejuni* infection [49]. IL-10^−/−^ mice (in C57BL/6j background) were held under specific pathogen free (SPF) conditions in the animal facilities of the Forschungseinrichtung für Experimentelle Medizin (Charité–Universitätsmedizin Berlin). To remove the commensal gut microbiota, mice were transferred to sterile cages and treated for 8 weeks with an antibiotic cocktail in the drinking water ad libitum containing ampicillin/sulbactam (1.5 g/L), ciprofloxacin (200 mg/L), imipenem/cilastatin (250 mg/L), metronidazole (1 g/L), and vancomycin (500 mg/L) as described previously [50]. Four days before infection, the antibiotic cocktail was replaced by curcumin (Sigma Aldrich, St. Louis, MO, USA; 0.5 mg/mL; solved in 2% Carboxymethyl cellulose (Sigma Aldrich, St. Louis, MO, USA) in phosphate-buffered saline (PBS; pH7.4; Sigma Aldrich, St. Louis, MO, USA)) in autoclaved drinking water. Mice were then infected via oral gavage with 10^8^ colony forming units (CFU) of *C. jejuni* strain 81-176 in a volume of 0.3 mL PBS. Six days after infection mice were sacrificed by isoflurane inhalation and colon samples were removed for tracer flux measurements analysis.

### 4.4. Ethics Statement

The animal experiments were carried out in our animal facility according to the German animal protection law (LaGeSo Berlin; approval number G0172/16, 13th Oct. 2016).

### 4.5. Electrophysiological Studies

Transepithelial electrical resistance (TER) was measured in vitro with a chopstick electrode pair under sterile conditions at 37 °C. The TER-values were corrected with the resistance of an empty cell filter and the bath solution. Colon samples from mice were mounted in modified miniaturized Ussing chambers (Institute of Clinical Physiology, Charité, Berlin, with an effective area of 0.049 cm^2^) in modified Ringer´s solution. Ringer´s solution for the Ussing experiments contained (in mM): NaCl (113.6), NaHCO_3_ (21), KCL (5.4), Na_2_HPO_4_ (2.4), MgCl_2_ (1.2), CaCl_2_ (1.2), NaH_2_PO_4_ (0.6), D(+)-glucose (10), D(+)-mannose (10) beta-hydroxybutyric acid (0.5), L-glutamine (2.5), and the antibiotics piperacillin (50 mg/L) and imipenem (4 mg/L), and was equilibrated for 15 min with carbogen gas to a pH of 7.4. One-path impedance spectroscopy measurements were performed as described previously [51] to delineate between epithelial and subepithelial conductance (G).

### 4.6. Epithelial Permeability

For permeability measurements, unidirectional flux studies were conducted with the paracellular marker fluorescein (332 Da; 100 µM) from the mucosal to the serosal compartment, either directly in 12-well plates or under short circuit current (I_SC_) conditions in Ussing chambers, as described earlier [7]. I_SC_ was recorded under voltage clamp conditions by an automatic clamp device (CVC6, Fiebig Hard & Software, Berlin, Germany) at 37 °C over 1.5 h. Fluorescein was dissolved either in Ringer´s solution or in media. Samples were taken every 15 min for one hour from the basolateral side, and fluorescence was measured in a spectrophotometer (Tecan GmbH, Maennedorf, Switzerland). Permeability was calculated from flux over concentration difference.

### 4.7. Cytometric Bead Array

Supernatants of co-cultures were collected 48 h after infection and analyzed using the Cytometric Bead Assay (CBA; BD Biosciences, Franklin Lakes, NJ, USA) according to manufacturer´s instructions (human Th1, Th2, Th17 Kit, Flex Set IL-1β) to determine the secretion of cytokines TNF-α, IL-1β, IL-6. Flow cytometric measurement were performed with FACS CantoII (BD Biosciences; Franklin Lakes, NJ, USA) and analyzed with FACP Array^TM^ software v3.0 (BD Biosciences, Franklin Lakes, NJ, USA).

### 4.8. Western Blot Analysis

For protein quantification, epithelial cells were washed twice with ice-cold PBS. Whole cell lysates were extracted with ice-cold lysis buffer. Whole cell lysis buffer was prepared with 150 mM NaCl, 10 mM Tris buffer pH of 7.5, 0.5% Triton *X*-100, and 1% SDS. A volume of 10 mL lysis buffer was supplemented with one Complete Protease Inhibitor Cocktail tablet (Roche AG, Basel, Switzerland). After lysis, cells were scraped from the filters, incubated for 60 min on ice, and vortexed every 10 min. After centrifugation (30 min, 15,000× *g* at 4 °C), the supernatant was collected. Sonification of the lysate followed by further centrifugation. Total protein quantification was performed by Pierce BCA assay (Thermo Scientific, Waltham, MA, USA) according to the product instructions using a Tecan plate reader (Tecan GmbH, Maennedorf, Switzerland) at an absorbance of 562 nm. Protein samples (10–20 µg) were mixed with 5xLaemmli buffer and loaded on a SDS polyacrylamide gel (for claudins 12.5%, for occludin 10% polyacrylamide). After electrophoretic separation of the proteins and transfer to a nitrocellulose membrane, membranes were blocked for 2 h at room temperature with 1% PVP-40 (Polyvinylpyrrolidone; Sigma Aldrich, St. Louis, MO, USA) in TBST/0.05%Tween-20 buffer. Primary antibodies anti-occludin (1:100; Sigma Aldrich, St. Louis, MO, USA), anti-claudin-1, -2, -4, -5, -7, -8 (1:100; Invitrogen, Carlsbad, CA, USA), and anti-β-actin (1:5000; Sigma Aldrich, St. Louis, MO, USA) as internal loading control were incubated overnight at 4 °C. Peroxidase conjugated secondary antibodies goat anti-rabbit IgG or goat anti-mouse IgG (Jackson ImmunoResearch, Ely, UK) were incubated for 2 h at room temperature. For protein detection, SuperSignal West Pico PLUS Stable Peroxide Solution (Thermo Scientific, Waltham, MA, USA) was used and signals were detected with Fusion FX7 imaging system (Vilber Lourmat Deutschland GmbH, Eberhardzell, Germany). Densitometric quantification was performed using ImageJ software 1.48v/Java 1.6.0_20 (Rasband, W. S., ImageJ, NIH, Bethesda, MD, USA), and the values were normalized to β-actin.

### 4.9. Immunofluorescence Staining

Cells grown on filters were rinsed twice with PBS and fixed for 30 min in 2% paraformaldehyde (PFA; Electron Microscopy Sciences, Hatfield, PA, USA) for immunostaining and microscopic analysis. Afterwards, the cells were washed twice with PBS, permeabilized with 0.5% Triton *X*-100 (Sigma Aldrich, St. Louis, MO, USA) for 7 min, and blocked for 10 min with 1% goat serum (Gibco, Carlsbad, CA, USA). The cells were incubated with the primary antibodies anti-claudin-4 and -8 (1:100; Invitrogen, Carlsbad, CA, USA), anti-ZO-1 (1:100; BD Biosciences, Franklin Lakes, NJ, USA) or cleaved caspase-3 (1:100; Cell Signaling Technology, Cambridge, UK) for 1 h at room temperature, followed by the secondary anti-rabbit or anti-mouse antibody for 1 h (1:500; Invitrogen, Carlsbad, CA, USA). Secondary antibodies were conjugated to Alexa-Fluor 488 or 594. The nuclei were stained with 4´-6-diamidino-2-phenylindole dihydrochloride (DAPI, 1:1000, Roche AG, Basel, Switzerland). Subsequently, the cells on the filters were washed with water and ethanol, then embedded in ProTaq Mount Fluor (Biocyc, Luckenwalde, Germany). The subcellular distribution of the tight junctions was analyzed by confocal laser-scanning microscopy (CLSM, Zeiss LSM780, Jena, Germany). Stained apoptoses with cleaved caspase-3 were counted microscopically.

### 4.10. Determination of Minimal Inhibitory Concentration Values

Determinations of minimal inhibitory concentration values (MICs) of *Campylobacter* strain 81-176 were performed in a broth microdilution assay. The wells of microtiter plates contained 2-fold serial dilutions of curcumin with a test range of 1–1024 µg/mL. For MIC determinations, inoculum level, growth medium, incubation time, and conditions were performed in accordance with the recommendations given in the Clinical and Laboratory Standards Institute (CLSI) document VET01-A4. In brief, *Campylobacter* colonies were taken from a blood agar plate and transferred into a tube containing 5 mL of 0.85% saline. The suspension was adjusted to the turbidity equivalent of 0.5 McFarland standard, and diluted 1:100 with growth medium. Subsequently, 50 µL of the diluted inoculum was added to each test well (50 µL) in the dilution series (containing substances at double the desired final concentration) and mixed. The microtiter plates were incubated for 24 to 48 h at 42 °C under microaerobic conditions to obtain sufficient growth. They were analyzed visually, and the lowest concentration preventing visible growth of bacteria was defined as the MIC. All susceptibility tests were performed in growth medium adjusted to pH of 7.4. *Campylobacter jejuni* reference strain DSM 4688 was used for quality control purposes. The MIC values of the quality control strain were determined in advance in three independent experiments using the broth microdilution and macrodilution method.

### 4.11. DNA-Uptake Assay

*C. jejuni* strain 81-176 was streaked out from a −80 °C stock and subcultured on Columbia blood agar containing 5% sheep blood (Oxoid, Thermo Scientific, Waltham, MA, USA). Plates were incubated overnight under an atmosphere containing 3.5% H_2_, 6% O_2_, 7% CO_2_, rest N_2_ at 37 °C. Cells were inoculated at OD_ini_ ~0.3 in sterile-filtered brain heart infusion broth (BHI; Oxoid, Thermo Scientific, Waltham, MA, USA), and incubated under the same atmosphere at 37 °C for 6–9 h at 140 rpm. Liquid cultures were subcultured under the same conditions in 5 mL BHI with or without 50 µM curcumin. A suitable OD_ini_ was chosen to reach optical densities of 0.1–0.6 after 17 h (± 3 h). 500 µL (± 300 µL) of the cell suspension was harvested by centrifugation (~16000× *g* for 5 min). The pellet was resuspended in 100 µL BHI with or without 50 µM curcumin. DNA labeling and uptake analysis were performed as previously described for *H. pylori* [52]. In short, *C. jejuni* BfR-CA-14430 genomic DNA was extracted using the PureLink Kit (Life Technologies, Thermo Scientific, Waltham, MA, USA), and labeled with fluorescein in a 1:1 (volume:weight) ratio of Label IT reagent to nucleic acid according to the manufacturer’s protocol (Mirus Label IT Fluorescein, Mirus Bio LLC, Madison, WI, USA). One µL of labeled DNA (100 ng/µL) was added to the cell suspension and incubated at 5% O_2_, 10% CO_2_, rest N_2_ for 30 min at 37 °C. Subsequently, cells were centrifuged (~16000× *g* for 5 min) and resuspended in 15 µL BHI supplemented with at least 3 U DNaseI (Roche AG, Basel, Switzerland). DNaseI digestion was performed at 37 °C for 5–10 min. The fluorescence microscope Axio Observer ZI (Zeiss, Jena, Germany) with a plan apochromatic 63*x*/1.4 objective and differential interference contrast (DIC) was used for the analysis of *Campylobacter* cells immobilized on 1.5% agarose pads. A metal halide light source (HXP120C) and a filter set with excitation at 470 ± 20 nm and emission at 525 ± 25 nm were used to visualize fluorescein. Exposing times varied between 250 ms and 750 ms. Images were taken by the 12-bit monochromatic AxioCam MRm camera. Cells with at least one fluorescent focus were considered active for DNA uptake, i.e., competent for natural transformation. For each condition, the fraction of competent cells was calculated and data are presented from three independent experiments.

### 4.12. Cytotoxicity

The CCK-8 assay (Cell Counting Kit-8, Thermo Scientific, Waltham, MA, USA) was performed in 96-well plates with colon epithelial cells HT-29/B6-GR/MR 1 day after seeding in accordance with the manufactures’ instructions. The cells were treated with different curcumin concentrations 2 h before infection. The absorption at 450 nm was measured by a spectrophotometer (Tecan GmbH, Maennedorf, Switzerland), and the percentage of viable cells calculated. In HT-29/B6-GR/MR cells, *C. jejuni* induced no significant loss in viability in a pre-test (control 100 ± 18% and DMSO control 81 ± 7% versus *C. jejuni*-infected 81 ± 4%, *n* = 3–4, *n*.s., one-way ANOVA with Bonferroni´s multiple comparison). No effect on cell viability was also seen at curcumin concentrations 30 µM, 40 µM, and 50 µM (85 ± 8%, 64 ± 4%, and 66 ± 6%, *n* = 3–4, *n*.s., one-way ANOVA with Bonferroni´s multiple comparison).

### 4.13. Ingenuity Pathways Analysis

Ingenuity Pathways Analysis (IPA, Qiagen Silicon Valley, Reswood, CA, USA) was used to evaluate curcumin-dependent expression data from a dataset from human colon biopsies, generated, and analyzed previously [6].

### 4.14. Statistical Analysis

All data are expressed as mean values ± standard error of the mean (SEM). Statistical analyses were performed with GraphPad Prism (version 7.0, GraphPad Software, Inc., San Diego, CA, USA) using one-way ANOVA with Bonferroni adjustment for multiple comparison or unpaired Student´s *t*-test. For data that were not normally distributed, the nonparametric Mann–Whitney *U*-test was used. *p* < 0.05 was considered to be statistically significant.

## Supplementary Materials

Supplementary materials are available online at https://www.mdpi.com/1422-0067/20/19/4830/s1.

## Abbreviations

BHI	Brain heart infusion broth
CBA	Cytometric bead array
CFU	Colony forming units
CLSM	Confocal laser-scanning microscopy
DAPI	4´-6-diamidino-2-phenylindole dihydrochloride
IFN	Interferon
IL	Interleukin
LPS	Lipopolysaccharide
MIC	Minimal inhibitory concentration
PBS	Phosphate buffered saline
PMA	Phorbol 12-myristate 13-acetate
TER	Transepithelial electrical resistance
TJ	Tight junction
TNF	Tumor necrosis factor
ZO	Zonula occludens

## Appendix A

### Acute Campylobacteriosis Contains Activated Signaling Pathways for which Curcumin Has Counter-Regulatory Properties in IPA Analysis

From acute infected *C. jejuni* patients a RNA-Sequencing (RNA-Seq) analysis with concomitant ingenuity pathway analysis (IPA, Qiagen Silicon Valley) was performed. Once gene expression modifications were revealed, a bioinformatic prediction about possible inhibitors of the changed gene regulation could be carried out [6]. The hypothesis is, that upstream regulators, which have an inhibited activation pattern, may re-activate in *Campylobacter* infection, when the substance is applied during infection. Consequently, inhibited upstream regulators could be protective or therapeutic approaches in *Campylobacter* infection by activation of the corresponding downstream pathways. Different potential candidates were screened for barrier-protective and anti-inflammatory properties in *C. jejuni* infection. One promising predicted regulator candidate that might counter-regulate the *C. jejuni*-induced downstream pathways was curcumin. Curcumin showed a significant effect on downstream target genes, with a *p*-value of 2.06E^−5^ and an activation ^z^-score of −3.489, and might therefore be another promising barrier-protecting or potential therapeutic substance in campylobacteriosis (Table S1). The *C. jejuni*-induced target genes in the dataset that could be counter-regulated by curcumin belong mainly to pro-inflammatory pathways, such as TNF-α or IL-1β. Another promising and studied candidate against *C. jejuni* infections is calcitriol (active vitamin D). Vitamin D shows in the RNA-Seq analysis with concomitant IPA analysis from patients in contrast to curcumin an even higher significance value (overlap *p*-value of 8.97E^−25^, z-score −6.25; with negative expression direction) [6].
