# Supplementary material for: Curcumin Mitigates Immune-Induced Epithelial Barrier Dysfunction by Campylobacter jejuni
Source: Int J Mol Sci. 2019 Sep 28;20(19):4830. doi: 10.3390/ijms20194830 (PMC6802366; doi:10.3390/ijms20194830)
Supplement: Supplementary file 1 [file ijms-20-04830-s001.pdf]

Appendix A

Acute Campylobacteriosis Contains Activated Signaling Pathways for which Curcumin Has Counter-Regulatory Properties in IPA Analysis

From acute infected *C. jejuni* patients a RNA-Sequencing (RNA-Seq) analysis with concomitant ingenuity pathway analysis (IPA, Qiagen Silicon Valley) was performed. Once gene expression modifications were revealed, a bioinformatic prediction about possible inhibitors of the changed gene regulation could be carried out [6]. The hypothesis is, that upstream regulators, which have an inhibited activation pattern, may re-activate in *Campylobacter* infection, when the substance is applied during infection. Consequently, inhibited upstream regulators could be protective or therapeutic approaches in *Campylobacter* infection by activation of the corresponding downstream pathways. Different potential candidates were screened for barrier-protective and anti-inflammatory properties in *C. jejuni* infection. One promising predicted regulator candidate that might counter-regulate the *C. jejuni*-induced downstream pathways was curcumin. Curcumin showed a significant effect on downstream target genes, with a *p*-value of $2.06E^{-5}$ and an activation *z*-score of -3.489, and might therefore be another promising barrier-protecting or potential therapeutic substance in campylobacteriosis (Table S1). The *C. jejuni*-induced target genes in the dataset that could be counter-regulated by curcumin belong mainly to pro-inflammatory pathways, such as TNF-α or IL-1β. Another promising and studied candidate against *C. jejuni* infections is calcitriol (active vitamin D). Vitamin D shows in the RNA-Seq analysis with concomitant IPA analysis from patients in contrast to curcumin an even higher significance value (overlap *p*-value of $8.97E^{-25}$, z-score -6.25; with negative expression direction) [6].

Table S1. Curcumin is an upstream regulator in *C. jejuni*-infected human mucosa identified by IPA

Upstream Regulator	Predicted Activation	Activation Z-Score	*p*-Value of Overlap	Target Molecules in Dataset
**curcumin**	inhibited	-3.489	$2.06E^{-5}$	ABCB1,ABCC1,ABCG1,ADAMTS4,ADIPOQ,APOE,ATOX1,AXIN1,BIRC3,BIRC5,CCNB1,CD44,CD80,CD86,CDK1,CDK4,CR1,CRP,CSNK1A1,CTGF,CXCL1,CXCL3,CXCL8,CXCR3,CXCR4,CYP2E1,CYP3A4,DDIT3,EDN1,EGFR,EIF3H,ERBB2,ETS1,FOS,FTL,GCLM,GFAP,GRIN2B,GRK6,HIF1A,HSP90B1,HSPA8,ICAM1,IFNG,IL17A,IL1B,IL6,JUN,LPL,LSP1,MMP1,MMP14,MMP3,MMP9,MTHFD1,NAMPT,NOS2,OLR1,PLAU,PPARGC1A,PRAP1,PRPS2,SELE,SERPINE1,SOCS1,SOCS3,SOD1,STAT3,TFAM,TFRC,TLR2,TLR4,TNF,TOP1,TOP2A,UBE2E2,UCP2,VEGFA,ZMYND8

ubcellular Tight Junction Protein Distribution in Co-Cultures in Confocal Laser-Scanning Microscopy

Curcumin alone showed no influence on the subcellular tight junction distribution of claudin-4 and claudin-8 in comparison to the untreated control (Figure S2).

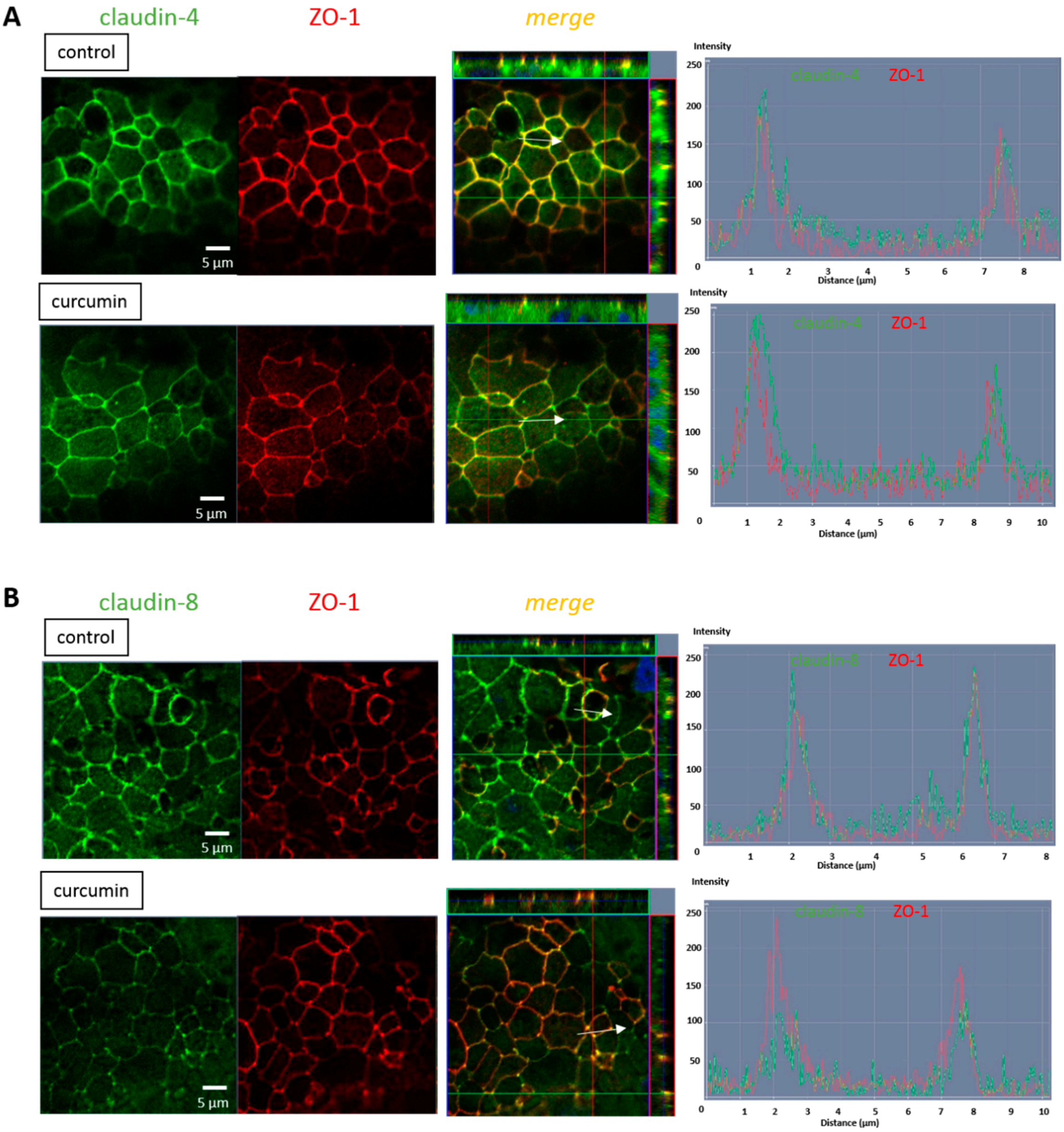

Figure S2. Tight junction distribution in control conditions after treatment without or with 50 µM curcumin. Representative confocal laser-scanning microscopy pictures of HT-29/B6-GR/MR after co-culturing together with immune cells. (**A**) Claudin-4 (green) and zonula occludens protein-1 (ZO-1, red), and (**B**) claudin-8 (green) and ZO-1 (red). Nuclei are stained in blue with 4′-6-diamidino-2-phenylindole dihydrochloride (DAPI).
